# Transverse testicular ectopia with persistent Mullerian duct syndrome: an operative eureka

**DOI:** 10.1016/j.ijscr.2020.04.035

**Published:** 2020-05-11

**Authors:** Muhammad Umer Mukhtar, Shehryar Ahmed Khan Niazi, Muhammad Zeeshan Sarwar, Syed Asghar Naqi

**Affiliations:** a King Edward Medical University/Mayo Hospital, Lahore, Pakistan; b Department of Surgery, King Edward Medical University/Mayo Hospital, Lahore, Pakistan

**Keywords:** Transverse testicular ectopia, Persistent mullerian duct syndrome, Ectopic testes, Male pseudo-hermaphroditism

## Abstract

**Introduction:**

Transverse Testicular Ectopia (TTE) is a rare entity in which both testes descend on the same side, and can be found in ectopic locations. When present with Persistent Mullerian Duct Syndrome (PMDS), a yet rarer entity, the persistence of Mullerian duct derivatives i.e. fallopian tubes, uterus, cervix and upper two-thirds of vagina occurs alongside testicular ectopia. There have only been about a hundred and fifty reported cases of TTE; a fifth of these accompanied by PMDS.

**Presentation of Case:**

Two middle-aged male patients presented with two separate complaints of inguinoscrotal swellings. In both patients, ultrasonography showed a hernial defect protruding into the scrotum on one side and the testis absent on the contralateral side. During hernia surgery, Mullerian duct remnants were found. Diagnosis of TTE with PMDS was established. Bilateral orchidectomy was done and Mullerian derivatives were excised.

**Discussion:**

There is controversy over the treatment of TTE with PMDS. Some authors,in addition to hernia repair, advocate the preservation of Mullerian structures because of risk to injury to vas deferens while others advocate resection of these structures due to risk of carcinoma. In pediatric patients, orchidopexy should be done to preserve fertility.However, in the older age group, orchidectomy should be done due to an increased risk of testicular carcinoma.

**Conclusion:**

TTE should be suspected in cases of unilateral inguinal hernia with contralateral undescended testes. Orchidectomy is recommended in patients older than 12 years old, otherwise, orchidopexy should be done. No Mullerian duct remnants should be left in situ.

## Highlights

• This case report accounts two remarkable cases of transverse testicular ectopia associated with persistent Mullerian duct syndrome.
• It gives a helpful discussion of the various treatment options for these two conditions when present alongside an inguinal hernia.
• It emphasizes the importance of resection of Mullerian duct derivatives and orchiectomy to eliminate the possibility of carcinoma.
• It highlights the importance of acute suspicion on part of the surgeon to diagnose the condition before taking to the operation theatre.

## 1 Introduction

Transverse testicular ectopia (TTE) is a rare condition that occurs solely in young males in which the same inguinal canal has the passage of both testes through it, in addition to the presence of an inguinal hernia [1]. The ectopic testis is present either in the contralateral scrotum, inguinal canal, or deep inguinal ring. Diagnosis is usually made during surgery for inguinal hernia associated with unilateral undescended testis as it usually presents as an inguinoscrotal hernia or a hydrocele. It is also associated with masked conditions like chromosomal anomalies, disorders related to sexual development and most importantly persistent Mullerian duct syndrome (PMDS) [2].

PMDS is a very uncommon form of internal pseudo-hermaphroditism in males, with genotype (46, XY) and phenotypically a young male. In this condition uterine, cervical and vaginal (upper half to two-third) structures form and persist as the derivatives of the Mullerian duct. Surprisingly not only testosterone is produced but there is normal responsiveness to it. Normally, there is regression of Mullerian ducts in male fetus due to suppression by the Mullerian inhibiting factor (MIF), secreted by the testes. Decreased levels of MIF or non-functionality of its receptors may be the mechanism behind persistent Mullerian duct syndrome [3].

Von Cenhossek, in 1886 discovered this condition, and Jordan in 1895 [3] while Nilson in 1939 described it with PMDS [4]. There are more than a hundred and fifty cases of TTE reported till date [5]. One fifth of these cases were accompanied by PMDS. Here, we describe a retrospective case report of two such cases which presented to Mayo Hospital, Lahore in 2019, in line with the SCARE criteria [6].

## 2 Case presentations

### 2.1 The first patient

**Fig. 1:**
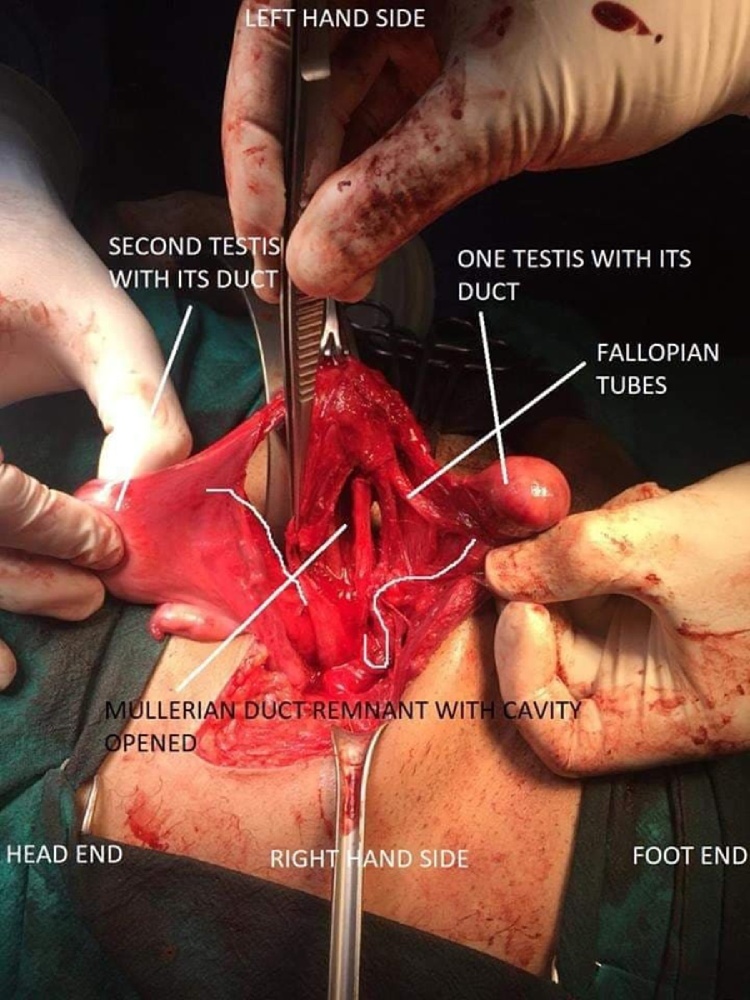
Both testes with PMDS.

A 32-year-old male presented with a swelling in the left inguinoscrotal region for 6 months, which gradually increased in size and became more prominent on standing. Patient had been married for the last 12 years with primary infertility. On examination, there was an ovoid, reducible, painless swelling in the left inguinal region. The left testicle was palpable in left hemi-scrotum with absence of the right testicle in right hemi-scrotum which was underdeveloped. Secondary sexual characters were normal. Baseline investigations were unremarkable. Ultrasonography showed a 1.9 cm hernial defect containing a piece of omentum and loop of bowel protruding into the left scrotum. Both testicles were reported on the left side. Provisional diagnosis of indirect incomplete left-sided inguinal hernia with transverse testicular ectopia was made. On exploration, rudimentary uterus (with an internal septum), cervix and bilaterally fallopian tubes were found. Both testis were present within hernia sac (Fig. 1). Bilateral orchidectomy was done. Mullerian remnants i.e. uterus, Fallopian tubes, cervix were also excised. The deep ring was closed and hernioplasty was done. Postoperative course and recovery of the patient were uneventful.

### 2.2 The second patient

**Fig. 2:**
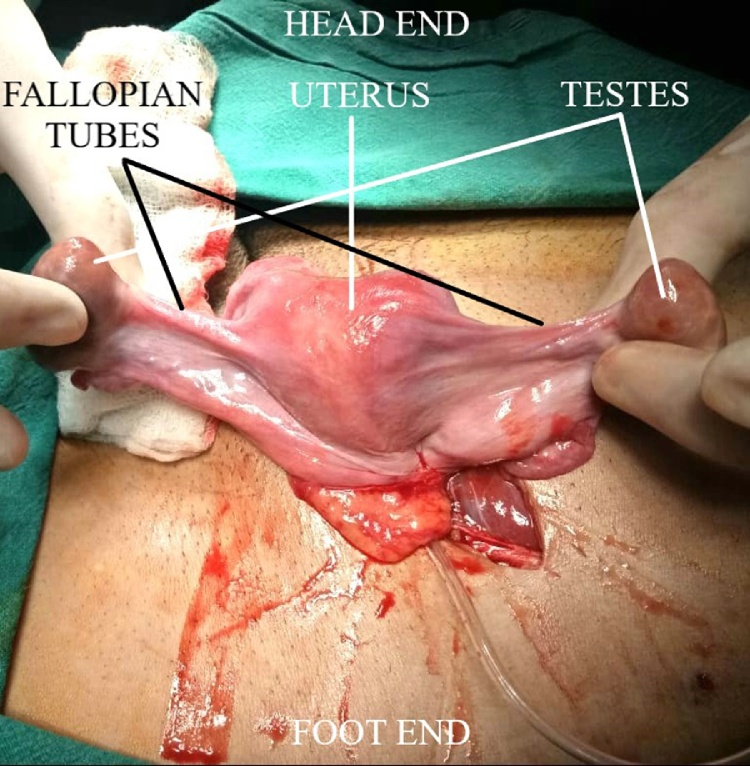
Both testes with Mullerian structures.

A 30-year-old male patient had history of a gradually enlarging swelling in the right inguinoscrotal region for several years. Physical examination showed a 5 × 5 cm globular, reducible, right inguinoscrotal swelling. Cough impulse was positive. Patient had a normal phallus and scrotum, with right testis palpable in right hemi-scrotum while the left one absent in the left hemi-scrotum. Secondary sex characters were normal. Baseline investigations were also unremarkable. Ultrasonography showed a hernia defect with contents protruding into right hemi-scrotum, with presence of both the testicles in the right inguinal canal. Provisional diagnosis of indirect, incomplete right-sided inguinal hernia with transverse testicular ectopia was made. On surgical exploration, bilateral fallopian tubes with their fimbriae, a well- developed uterus and superior two-third of vagina were found alongside the hernial sac that contained both right and left testicles. (Fig. 2) Bilateral orchidectomy and excision of abnormal Mullerian structures was done. Deep ring was closed and hernioplasty was done. Postoperative course and recovery were unremarkable.

## 3 Discussion

Normally both the testicles descend to the scrotum at birth, the failure of which causes the "undescended testes". The sites reported for undescended testes are the external inguinal ring, inguinal canal, deep ring, femoral triangle, near the penile base, supra-pubic region and around the perineum [1,2]. If both testes are in the same inguinal canal, i.e. one of the testes has migrated to the other side, it is known as TTE. The exact cause of it is unknown but there are many factors and theories regarding it; some postulate about both testes having developed from the same genital ridge while others think that due to adherence in Wolffian ducts, one testis is dragged along by the other in the later one's path of descent [1]. In PMDS, the uterine, cervical and vaginal derivatives may be either developed or rudimentary [5]. The causative factor may be the inability to produce Mullerian Inhibitory factor, or its defective releasing mechanism or timing. The decreased effect of properly produced MIF on tissues can also be a probable cause [1]. The patient has normal masculine secondary sexual characters, male external genitalia, normal facial and pubic hair, normal penile development and erection, with androgen levels being in normal limits [7,8]. However, due to prolonged ectopic location of testes, there is presence of testicular atrophy on microscopy. Even in patients with presence of spermatozoa, fertility is a rarity due to the defects in their motility [5].

TTE can be classified according to the anomalies accompanied by it. Type I has an inguinoscrotal hernia only (40 %–50 %). Type II is associated with PMDS (20 %–30 %) as in our cases [9]. Type III is a rare entity, which has scrotal abnormalities, cysts in seminal-vesicles, horseshoe kidney, hypospadias and common ductus deference associated with it [1,7].

It usually presents around four years of age. During hernia surgery or surgery for cryptorchidism, this condition is most commonly detected, that too, incidentally [4,9]. TTE is usually associated with an inguinoscrotal hernia on one side along with an impalpable testis on the opposite side. Similar was in our cases except the patients were in the fourth decade of life. Therefore, every surgeon performing surgery on an inguinal hernia should be well aware of encountering this condition. It is difficult to diagnose it preoperatively due to the rarity of the disease. Preoperative imaging could be of help if there is a strong suspicion of the condition.

Preservation of fertility and the placement of testis in hemi-scrotum is the best treatment option in young males. Literature shows that the incidence of testicular cancer peaks to 6 times if the surgical procedure of orchidopexy is delayed until after the age of 10–11 years [2,8,10]. In our cases, patients were 30 and 32 years old and chances of malignant transformation were high so bilateral orchidectomy was done, which is recommended in the literature [7,8].

There is a considerable controversy over the optimal surgical treatment of persistent Mullerian duct syndrome. Certain surgeons recommend the preservation of the Mullerian derivatives at all [1] as there is a possibility of iatrogenic injury to the ductus deferens. On the other hand, it's now known that these persistent Mullerian derivatives have a high risk of developing cancer, especially in the cervix. Hence exicion of uterus, cervix, fallopian tubes and upper two-thirds of vagina must be done to avoid future development of carcinoma [5]. In our case, resection was done from the uterus to upper two-thirds of the vagina till the proximity of the urethra.

## 4 Conclusion

Being a very rare disorder, transverse testicular ectopia has a pathology that is not clear and still subject to debate. Diagnosis of transverse testicular ectopia should be suspected if there is presence of a unilateral inguinal hernia in association with a contralateral undescended testicle. The surgical option of bilateral orchidectomy can be considered if the patient’s age is more than 12 years, otherwise, an attempt to preserve fertility via orchidopexy should be made. Authors recommend that Müllerian duct remnants should not be left in situ if such an approach is used.

## Declaration of Competing Interest

No conflict of interest.

## Funding

No funding sources were utilized.

## Ethical approval

Ethical approval was taken from the Head of Department.

## Consent

Informed written consent was taken from both the patients for publication of this case report.

## Author contribution

Muhammad Umer Mukhtar: Data Curation, Writing – original draft, Writing - Review & Editing, Project Administration

Shehryar Ahmed Khan Niazi: Surgeon, Writing - review & editing, Resources

Muhammad Zeeshan Sarwar: Conceptualization, Writing - review & editing, Supervision

Syed Asghar Naqi: Supervision

## Registration of research studies

Registry Name: UMIN-CTR (University hospital Medical Information Network - Clinical Trials Registry).

UIN: R000045229UMIN000039655.

## Guarantor

Shehryar Ahmed Khan Niazi.

## Provenance and peer review

Not commissioned, externally peer-reviewed.
